# Barriers and facilitators to mental health promotion for Mexican immigrants in the U.S. through the Ventanillas de Salud program

**DOI:** 10.3389/fpubh.2023.1268253

**Published:** 2023-09-28

**Authors:** Inés González Casanova, Delia Lilian Martínez Rodriguez, Julissa Ortiz Brunel, María Gudelia Rangel Gómez, Mary de Groot, Alicia Fernández

**Affiliations:** ^1^Department of Applied Health Science, School of Public Health, Indiana University Bloomington, Bloomington, IN, United States; ^2^Oaxaca Ministry of Health, Oaxaca, Oaxaca, Mexico; ^3^Department of Sciences of Human Movement, University Center for Health Sciences, Universidad de Guadalajara, Guadalajara, Guadalajara, Jalisco, Mexico; ^4^Comision de Salud Fronteriza Mexico-Estados Unidos, Tijuana, Baja California, Mexico; ^5^Division of Internal Medicine, Indiana University School of Medicine, Indianapolis, IN, United States; ^6^San Francisco General Hospital, University of California, San Francisco, San Francisco, CA, United States

**Keywords:** mental health promotion, mexican immigrants, Latino mental health, depression screening, implementation science, health disparties, COM-B, Theoretical Domains Framework

## Abstract

**Introduction:**

Mental health promotion and screenings are recommended as part of standard preventive care. Mexican immigrants in the U.S. are at high risk for mental health illness especially after the COVID-19 pandemic, however access to mental health prevention for this population has been limited, which results in important implementation and equity gaps. The Ventanilla de Salud (VDS) program provides preventive services through Mexican consulates in the U.S.

**Objective:**

The objective of this study was to assess capability, opportunity, and motivation for promotores to implement mental health programming through the VDS, leveraging early experiences of ongoing mental health prevention efforts.

**Methods:**

This was a qualitative study using the Capability, Opportunity, and Motivation for Behavior Change model (COM-B). We conducted 9 focus groups with 40 VDS *promotores* and 6 semi-structured interviews with program stakeholders. Data were analyzed using inductive and deductive coding.

**Results:**

We found high levels of interest from the leadership, partners, and *promotores* to provide mental health services through the VDS. Early implementation of a mental health strategy that included training sessions for *promotores* and mental health promotion, screenings and referrals for VDS users was ongoing. We identified facilitators and barriers that could affect capability, opportunity, and *motivation* to provide mental health services. Facilitators included promotores’ extensive knowledge about the importance of mental health, *promotores* service mindset and commitment to provide services to VDS users, and general support from the VDS network and partners. Barriers included *promotores*’ turnover, need for additional economic compensation, burnout, competing priorities, and lack of mental health professionals to provide clinical services or supervision. Additional investments are recommended to support *promotores*’ well-being.

**Conclusion:**

The main lesson learned from this study was that investing in VDS *promotores*’ training, resources, and well-being is key to their capability, opportunity and motivation to provide mental health services for Mexican immigrants in the US. Results from this study can be applied to improve the ongoing VDS mental health strategy and increase its impact on the mental health of Mexican immigrants.

## Introduction

Mental illness is the leading cause of disability worldwide (1). Mexican immigrants, the largest foreign-born group in the United States, are at higher risk of developing depressive and anxiety disorders compared to non-migrant Mexicans and to their US-born counterparts (2). It is estimated that almost 40% suffer from anxiety disorders and almost 15% have been diagnosed with depressive disorders, which is probably an underestimation due to the high prevalence of undiagnosed mental illness in this population (3). Hence, it is essential to implement evidence-based interventions to prevent mental disorders among Mexican immigrants.

The World Health Organization has identified mental health promotion and screening for mental disorders as essential for the prevention of mental illness (4). However, Mexican immigrants in the US face barriers in access to mental health preventive services and care (3). For instance, Mexican immigrants are less likely to receive health screenings for mental disorders or seek mental health treatment compared to other Hispanics in the US (5). National US data show Mexican immigrants have, on average, fewer years of education, lower income, and are less likely to have health insurance than US-born individuals, other Hispanics and immigrants from other countries (6–8). Language and structural barriers also complicate access to preventive care in this population (9). To address these many barriers, a promising approach is to incorporate mental health promotion and screening into existing health prevention initiatives that already reach this at-risk population (10, 11).

The Ventanilla de Salud (VDS), implemented through the network of Mexican consulates, is an initiative that already reaches a large number of Mexican immigrants throughout the US. The VDS plays a critical role in the preventive care of recent immigrants, providing health education, cardiometabolic risk screenings, and referrals to community resources or healthcare to millions of Mexican and other Latino immigrants that would otherwise not have ready access to these services (12). According to the latest evaluation of the program in 2020, the VDS provided screenings for cardiometabolic risk factors (hypertension, hyperglycemia, dyslipidemia, and overweight) to more than 500,000 Latino immigrants in 2019 (13). While the specific impact of the VDS screening program on health outcomes has not been evaluated, screening for cardiometabolic risk factors in combination with appropriate referrals is an evidence-based intervention has been shown to increase preventive behaviors, improve quality of life, and linkage to care (14). Taken together, this evidence supports an important contribution of the VDS to the cardiometabolic health of Latino immigrants in the US.

Since 2018, the VDS began implementing a strategy to integrate mental health promotion into their existing services through basic training for *promotores*. More recently, during the COVID pandemic, this effort was expanded to include screenings and referrals (3). We conducted this study to evaluate existing efforts to provide mental health prevention services through the VDS network and to identify barriers and opportunities for additional mental health programming (screening and services) through the VDS.

## Methods

### Study design

This was a qualitative study aimed at assessing the implementation of mental health strategies through the VDS program. It followed the capability, opportunity, and motivation to affect behavior (COM-B) model and the Theoretical Domains Framework (TDF) (See a more detailed description of COM-B and TDF below). A description of the study following the consolidated criteria for reporting qualitative studies (COREQ) (15) is presented next.

### The VDS program

The VDS is a program funded by the government of Mexico and implemented in the US to facilitate access to primary and preventive health services to Mexican immigrants. There are currently 49 VDS and two mobile units operating in the Mexican consular network in the US (13). According to information from the Mexican Ministry of Health, between 2013 and 2018, the VDS program served over 9 million people, providing more than 25 million individual services (8). More recently, in the period from January 2019 to June 2021, 14 million services were provided to 5 million people (13).

The VDS program is implemented through a health *promotores* model, where each Ventanilla has a team of one to four *promotores* and a coordinator, as well as a local non-for-profit partner responsible for managing each site. This team works together at a VDS to provide high quality health information, health education, advice, and referrals in a safe environment, with the goal of improving the health and quality of life of Mexican immigrants (16). *Promotores* background and education varies but some of the qualifications for the job include college education preferably in a health related field, or training as community health worker, community outreach worker, or health promotor; experience working with Latino families; basic knowledge about the health care system and social services; experience conducting outreach in diverse settings; and experience providing community education. There is also a national coordination center based in San Diego that is responsible for establishing partnerships in the US and for overall program management and evaluation. The coordination center works closely with the Mexican Ministry of Health and the Mexican Institute for Mexicans in the Exterior, which are the two governmental organizations responsible for the program in Mexico.

### Existing mental health services implemented through the VDS

A VDS mental health strategy is already underway where training sessions in the WHO mental health Gap Action Programme (mhGAP) intervention guide, which is an integrated package of mental health interventions, the development and implementation of a COVID-mental health screening questionnaire, and a telephone line to provide psychological services in Spanish to people who completed the screening questionnaire and were deemed at risk. In addition, VDS *promotores* developed a database to generate a network of local partners in mental health.

Training in the WHO mental health Gap Action Programme was conducted in collaboration with the Mexican section of the United States-Mexico Border Health Commission (CSFMEU) and the Pan American Health Organization (PAHO) and targeted VDS health personnel and community *promotores*. This was done to strengthen technical capacities in mental health through basic training for detecting and referring patients with mental health problems. Forty-two health *promotores* were initially trained in 2018, and some updates were made available as new *promotores* joined the VDS.

In 2020, as a response to the COVID-19 pandemic, the screening questionnaire and the psychological services phone line were implemented through a collaboration between the Mexican Ministry of Foreign Affairs, the Mexican Ministry of Health, the Mexican Section of the CSFMEU, the Migrant Clinicians Network (MCN), and the National Autonomous University of Mexico (UNAM) with the objective of providing remote mental health services in Spanish. At the time of this qualitative study, sixty-one Mexican immigrants had been given a questionnaire to determine if they had mental health needs. Among those, forty-two people consented to be contacted by professionals from the Faculty of Psychology of UNAM. The problems identified mainly were anxiety and depression, followed by substance abuse and stress (3).

### Theoretical framework

This study was guided by the Capability, Opportunity, and Motivation behavioral change model (COM-B), that recognizes behavior as a part of an interacting system involving all these components (8, 17). The target behavior in this case was for promotores to conduct mental health screening, education, and/or referrals during VDS visits. We also used the Theoretical Domains Framework (TDF) (18) to operationalize the COM-B model. The TDF is an implementation framework developed by behavioral and implementation scientists. It operationalizes behavior into 14 theoretical constructs that can be mapped to capability, opportunity, and motivation (see Table 1). The use of COM-B and TDF has been recommended to collect information about barriers and opportunities that then can lead to the development and implementation of individual behavioral change interventions (17). In this study, we used the COM-B and TDF to develop the data collection instruments following the process recommended by Michie et al.. Similarly, deductive codes were based on the TDF framework (Table 1).

**Table 1:** TDF domain definitions^*^, questions, and testimonies from VDS promotores and key actors.

COM-B	TDF domain	TDF domain questions (interview guide)	Testimonies
*Capability*	Knowledge: An awareness of the existence of something. Including knowledge of condition/scientific rationale; procedural knowledge; knowledge of task environment. Cognitive and interpersonal skills: An ability or proficiency acquired through practice. Memory, attention, and decision processes: The ability to retain information, focus selectively on aspects of the environment and choose between two or more alternatives. Behavioral regulation: Anything aimed at managing or changing objectively observed or measured actions.	What are the most important mental health problems to address in the VDS? What knowledge do you think could be important for VDS promotores to identify mental health problems in users? What skills related to mental health do promotores have? Do they need additional skills or training? What tools, questionnaires or tests are available in the VDS to for promotores to screen and identify mental health problems in users? What do the VDS promotores usually do if they identify a mental health problem in users? How can the mental health strategy be incorporated into the work routines of the VDS promoters? How can we take advantage of the existing work routines in the VDS to facilitate new tasks related to mental health screening?	*“Yes, we also went through the mhGAP guide and we had the full training and then feedback with case studies. And well, it is a practical guide, maybe not all cases apply to us, but it is always good to know and learn. By the time we have the opportunity, we are already trained” (promotor, focus group)* *“Sometimes, the emotional health course at UNAM stressed me more because I did not have the time and had many activities to do. So, I had to talk to the psychologists from UNAM, and they told me that I was fine, not to worry” (health promoter, focus group)* *“At the beginning of the year, when we had to close the Ventanilla [because of the COVID-19 pandemic], we interacted with people through telephone calls. I felt a little helpless and guilty that I could not be there with people. To improve that aspect, we began to do surveys where people could express a need, and I talked to them and made their referral, but it was frustrating that I could not be there because they had several needs, including mental health. […] and now I feel perfect that I can interact with people, obviously with the preventive measures, but there was a difficult moment, I was doing my best for them when there were limitations” (promotor, focus group)* *“For us it is very important to know how to listen to them and simply listen to the person, when they tell you -I am not interested that my glucose level is at 250, 300. I am not interested that my family is going through this-. But you know that this person has a problem and it’s important to refer them. If the person is not taking control of their emotional health, this is going to get worse and worse” (promotor, focus group)*
*Opportunity*	Environmental context: Any circumstance of a person’s situation or environment that discourages or encourages the development of skills and abilities, independence, social competence, and adaptive behavior. Resources: Material resources, barriers and facilitators. Social influences (norms): Those interpersonal processes that can cause individuals to change their thoughts, feelings, or behaviors.	Are there competitive tasks that complicate implementing the mental health strategy in the VDS? If yes, which ones? To what extent do physical or resource factors facilitate or hinder the ability of VDS promotores to carry out a mental health strategy and referrals? To what extent could work teams be created among the VDS to implement the mental health strategy? Is there any social support for promotores to implement the mental health strategy? If yes, please describe.	*“Lack of time, that is the first concern, excessive workloads, excessive demands from the consulates, that you have to deliver numbers and you leave mental health aside.” (key actor, semi-structured interview)* *“[Promotores]go out of their way to help the person if they see that they need something. So, if they see that a person arrives very badly … the first thing they do is be empathetic. [Our role] is trying to support them, give them resources, skills” (key actor, semi-structured interview)* *“… We have allies who come to our VDS to provide free counseling with through the “Healthy mind, healthy life” program. Then we can not only measure blood pressure, glucose, but also integrate mental health” (health promotor, focus group)* *“Stigma, discrimination, and social exclusion are definitely the worst enemies. We have people with a severe risk of mental health who do not receive specialized services and are discriminated against in the educational, social, and health fields. So, stigma is the biggest enemy of mental health that we need to defeat.” (key actor, semi-structured interview)*
*Motivation*	Social/Professional role and identity: A coherent set of behaviors and displayed personal qualities of an individual in a social or work setting. Beliefs about own capabilities (Self-efficacy): Acceptance of the truth, reality, or validity about an ability, talent, or facility that person can put to constructive use. Belief about consequences: Acceptance of the truth, reality, or validity about outcomes of a behavior in a given situation. Goals and motivation: Mental representations of outcomes or end states that an individual wants to achieve. Emotion: A complex reaction pattern, involving experiential, behavioral, and physiological elements, by which the individual attempts to deal with a personally significant matter or event. Nature of the behaviors: Routine / automatic habit, breaking habit, direct experience, and representation of tasks	How could your job position contribute to the design and implementation of a strategy to address mental health issues in the VDS? How difficult or easy would it be for you to direct or coordinate mental health promotion and services with the VDS promotores? Why? Do you think that implementing a mental health strategy through the VDS would help people improve their health? Why or why not? Do the benefits of carrying out a mental health strategy in the VDS outweigh the consequences? How much do you want to promote mental health care in VDS users? What would serve as an incentive for promoters to overcome these competing factors? Have the VDS promoters been made aware of mental health issues? Have you identified any emotional responses from promotores as they implement the mental health strategy?	*“The Ventanillas’ promotores have always been very empathetic to the needs of the people; that is, I believe that if you want to be a promotor, you have to have a big and golden heart to be able to help.” (actor key, semi-structured interview)* *“It is important to take care of the mental health of the promoters since providing mental health depends on them; that’s where we start.” (key actor, semi-structured interview)* *“…Improve economic compensation, I think they will appreciate that and tools to be able to do their job better. Other incentive that they are always looking for is a type of certificate, for example, when we give them a webinar, they love that it has some kind of certificate” (key actor, semi-structured interview)* *“I think that we are going to feel bad with the cases that come to the Ventanilla, but I believe that feeling bad, feeling sad, or wanting to cry with them is part of the empathy that we must have with them” (promotor, focus group).* *“*(Speaking about the COVID and mental health screening questionnaire) *The only thing I would also say is that many of the questions are about COVID, and not all cases are going to be depression or anxiety due to COVID; that is, people have many other problems, and COVID is not a priority. So, it’s confusing, but it does help, because as I told you, it helps us to see more of the symptoms that they are experiencing and, obviously, that also allows us to make the referral” (promotor, focus group)*

^*^Definitions obtained from Atkins et al. (18) and Michie et al..

### Data collection

The qualitative data collection was conducted between July and December of 2021 through interviews with key actors and focus groups with health *promotores*. The semi-structured interview and focus group guides were developed based on the COM-B model and the TDF framework (Table 1) and pilot-tested with former and current VDS employees (*n* = 3). All data collection activities were conducted in Spanish by native Spanish speakers.

The field psychologist (DLMR) conducted six semi-structured interviews with key actors from the VDS program including the national VDS coordinator, other program administrators, and collaborators from the National Autonomous University of Mexico (UNAM) who had been responsible for implementing mental health strategies through the VDS. Information obtained from key actor interviews was complemented with bibliographic research and available publications (often suggested by the stakeholders) to better understand the context and previous efforts to provide mental health services through the VDS program.

In addition, nine focus groups were conducted (led by DLMR, with IGC or JOB present for support and note-taking) with *promotores* who worked in VDS throughout the United States. Focus groups were divided by the following regions: East [2], West [2], Center [1] and Border [2]. Additionally, two groups with *promotores* from any region who could not attend the scheduled groups were conducted. The number of participants in each group varied between 3 and 6.

### Study participants

We used intentional selection to choose the key actors who were interviewed (VDS strategy coordinators and administrators and UNAM collaborators). For the focus groups, an e-mail was sent to all VDS health *promotores* and site coordinators inviting them to participate in the study. Calendly was used to allow *promotores* to sign up to their preferred groups. Participants were compensated for their participation with a 25 US dollar gift card. The criteria used were being 18 or older, working as *promotores* or coordinators in a VDS, and giving their verbal consent to participate in the study.

### Data analysis

We performed content analysis with inductive and deductive coding. Audio recordings of the interviews and focus groups were transcribed by JOB. The coding guide was developed by IGC based on TDF domains and refined through several discussions with DMLR, and JOB. We used inductive coding by looking at emerging patterns, and deductive coding guided by COM-B and the TDF framework. An initial subset of an interview and a focus group was coded for reliability purposes and the coding guide was refined until a kappa of 0.82 was reached. NVivo 14 qualitative data analysis software was used. Analyses were conducted in Spanish by native speakers and then the results and quotes were translated into English while writing this manuscript. Quote translations were reviewed for meaning by bilingual (Spanish and English) speakers.

## Results

In total six VDS key actors participated in the semi-structured interviews and 40 *promotores* participated in the focus groups. *Promotores* were on average 46 years, had been working in the VDS for almost 5 years, and were primarily women (Table 2).

**Table 2:** Sociodemographic characteristics of Ventanilla de Salud promotores who participated in focus groups (*n* = 40).

Characteristics [mean ± SD or %(*n*)]	*N* = 40
*Age (years)*	46.2 ± 10.6
20–30	5.0 (2)
31–40	22.5 (9)
41–50	37.5 (15)
51–60	22.5 (9)
61–70	10.0 (4)
Did not answer	2.5 (1)
*Time working at VDS*	4.4 ± 5.8
Less than a year	15.0 (6)
1 to 5 years	35.0 (14)
More than 5 years	50.0 (20)
*Sex*	
Female	85.0 (34)
Male	15.0 (6)
*VDS region*	
Border	25.0 (10)
Central	35.0 (14)
West	7.5 (3)
East	32.5 (13)
*Ethnicity*	
Hispanic/Mexican/Latino/Chicano/Mestizo	95.0 (38)
Did not answer	5.0 (2)

### Capability, opportunity and motivation to provide mental health services through the VDS

**Figure 1:**
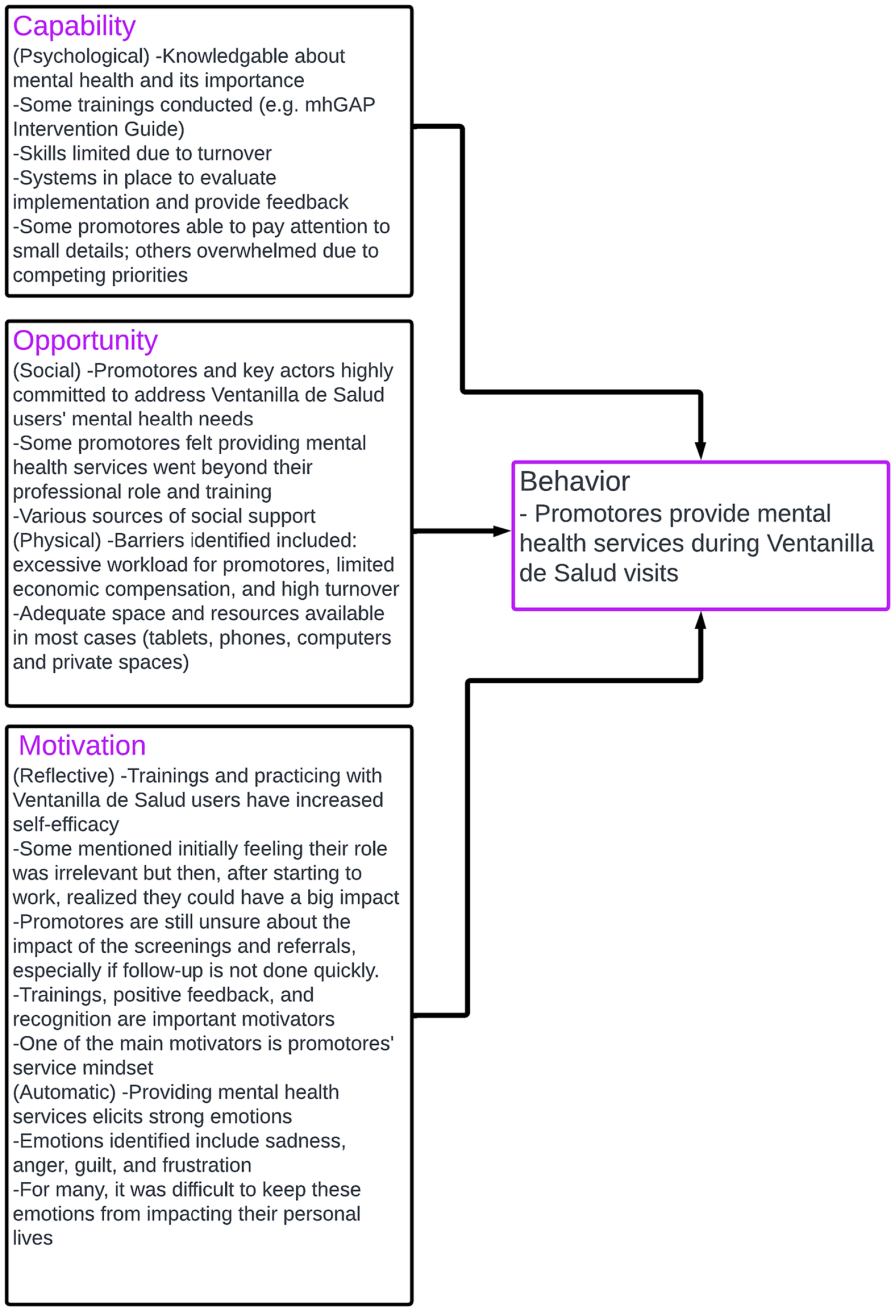
Summary of main results for capability, opportunity, and motivation of Ventanillas de Salud *promotores* to provide mental health services.

The main findings for the capability opportunity and motivation domains are summarized in Figure 1. The detailed explanation by TDF domains, sample interview questions, and quotes are included in Table 1.

#### Psychological capability

For psychological capability, *promotores* expressed basic knowledge about mental health and its importance, especially for VDS users. The *promotores* repeatedly expressed their goal of providing mental healthcare to VDS users from a holistic perspective.

“To me, mental health is an integral part of our health. Many times, we focus on physical [health] and leave mental health behind because we see it as a taboo. Then, what we talk with the community is that mental health is part of our wholesome health.” (Promotor, focus group).

This was potentially due to the ongoing training opportunities provided by the VDS leadership team, as well as to their interactions with VDS users in need of these services.

“Yes, we also went through the mhGAP guide and had the whole training and then feedback with practical cases. And well, is a practical guide, maybe not all the cases are applicable for us, but it is always good to know and learn. For whenever we get an opportunity, we are already trained.” (Promotora, focus group).

However, despite these training sessions, there was a general perception that skills needed to conduct mental health screenings and referrals remained limited due to the high turnover in *promotores*.

Ongoing supervision and feedback were potential factors that can improve the implementation of mental health screenings and referrals through providing behavioral regulation.

In terms of attention and decision processes, many *promotores* mentioned that they were able to listen attentively to users and to pay attention to small details, however, some *promotores* said that they felt overwhelmed due to competing priorities, and sometimes were not able to provide these services.

#### Social opportunity

For social opportunity, *promotores* expressed mixed feelings related to the question if providing mental health services was part of their professional role. Some saw themselves as highly empathetic, with great commitment to and interest in providing these services that they see as a priority for VDS users. However, others felt that the training sessions went beyond their role of *promotores* because they included information about the diagnosis and treatment of mental health illnesses and most *promotores* do not have formal training or credentials in mental health.

“My concern when I heard what they were doing was exactly what (person 1) was talking about. I do not think it is adequate that the promoters of the Ventanillas, who do not have professional training in mental health, are the ones who lead this type of situation.” (promotora, focus group).

Similarly, the *promotores* identified several sources of social support for them including the network of external organizations and partners to the VDS, as well as the collaboration with the National Autonomous University of Mexico, their fellow *promotores*, and the national managers of the VDS program.

Stigma and discrimination associated with mental illness were identified as social norms that need to be addressed, and *promotores* already started to work on some initiatives. Similarly, gender roles were also identified as an important consideration, including the problem of machismo.

#### Physical opportunity

Regarding opportunity, the *promotores* expressed that resources are generally adequate to provide mental health services. However, they also identified various environmental stressors including excessive workload, lack of time, high turnover of *promotores*, increased workload without increased compensation, time consuming data entry requirements into a monitoring system, lack of support from mental health professionals, and the need to shift activities online during the COVID-19 pandemic.

“Lack of time, that is the first issue, excessive workloads, excessive demands, that you have to report numbers, and you leave mental health aside. … that could mean that they do not have time to do this” (key actor, semi-structured interview).

### Reflective motivation

For reflective motivation, we found that most *promotores* felt increasingly capable of listening to mental health concerns, providing general information and referring VDS users as needed. They explained that this increase of self-efficacy has been built through the training sessions and the practice of these skills with users.

In general, *promotores* and stakeholders feel that the actions they take providing mental health services through the VDS will positively impact the well-being of the VDS users. However, a challenge they have found witnessing the impact of their actions is that often VDS users do not return to the VDS for years and *promotores* never know if they are adequately linked to mental health services. Even when *promotores* referred users to VDS programs, such as the phone line staffed with psychologists from the National University of Mexico, they never know if the users received the call backs as planned or if the connection was lost. Some *promotores* expressed concern that maybe the psychologists are not being able to connect with the users, and they never received the mental health care they needed. This concern sometimes affected their motivation to conduct the screenings.

Both stakeholders and *promotores* identified the latter’s attitude towards service as the most important motivation for incorporating mental health services. However, stakeholders also highlighted the importance of improving economic incentives, giving recognition to the *promotores*’ work and providing constant feedback as motivators for *promotores* to continue providing the services.

“We need to provide economic motivation[to the promotores], I think they will appreciate that and tools to be able to do their job better, they are always looking for that and that these tools have a type of certificate, for example, when we give them a webinar, they love that it has some kind of certificate that they took an hour to practice this and this” (key actor, semi-structured interview).

#### Automatic motivation

Regarding automatic motivation, a theme that was constantly highlighted by *promotores* was the strong emotional response that they experience when providing mental health services. They identified sadness, anger, guilt, and frustration as the main emotions felt when providing mental health services through the VDS. They mentioned feeling afraid of the magnitude of the responsibility that helping users with their mental health problems represents. In some instances, the stress related to providing these services started during the training. Others described how the COVID-19 pandemic also generated a mental health emergency in the VDS users, and *promotores* were left to address it with limited tools. In some cases, this led to compassion fatigue in the *promotores*, where they started to take on the users’ emotions. To address this, VDS leadership and mental health partners implemented some self-care workshops that the *promotores* described as very helpful. In terms of habits done automatically and routinely, *promotores* identified that there was a setback during the COVID-19 pandemic because all the normal procedures had to be adapted to fit the new reality. They were able to adjust some of the procedures to continue to provide mental health information, screenings, and referrals to some users.

“I think that we are going to feel bad with the cases that come to the Ventanilla, but I believe that feeling bad, feeling sad, or wanting to cry with them is part of the empathy that we must have with them” (health promoter, focus group).

### Emerging codes

The following sections describe codes that were identified inductively during the data analysis phase.

### Mental health activities independently designed and implemented by *promotores*

Besides the strategies designed by the VDS in partnership with the Mexican National Autonomous University (UNAM) psychology team, *promotores* designed, adapted and implemented diverse approaches to provide mental health services, in response to the specific needs of the VDS users in their consulates. Some of these approaches included: providing mental health education through videos, WhatsApp messenger, Facebook lives, virtual and face to face talks; following up with users through text messaging or phone calls to see if they had received the care that they needed; agreements with local organizations or with volunteer mental health professionals; virtual Zumba dance, yoga or other fitness classes; and having a box with different stress balls, plush toys and other trinkets to release anxiety or stress. Some VDS relied on psychology students doing their internships to provide mental health preventive services.

“It would take all the publicity in the world. Right now, we have a poster that says mental health support; come to your Ventanilla. This, I think, could be educational videos. I think that what works the most are social networks so that videos can be handy, and posts on social networks, on Facebook Live, on the Facebook of the Mexican consulate, of the Ventanilla de Salud.” (promotor, focus group).

### Self-care strategies employed by health *promotores*

Self-care was a recurring topic that was mentioned by both key actors and *promotores* as essential to sustaining the mental health strategy and supporting *promotores*. Key actors introduced the context of self-care through formal training and virtual sessions during the COVID-19 pandemic. The *promotores* quickly adopted these strategies and used them to deal with the emotional load of providing mental health services to the VDS users. Self-care strategies mentioned by the *promotores* included using oils, aromatherapy, lime and chamomile tea, Himalayan salts, music, yoga classes, mindfulness, psychotherapy, relaxation exercises, and activities such as going out for coffee and walking around. They mentioned the need to create an emotional support group for them.

“I went to therapy after so many deaths with COVID, dead and dead and dead, and obviously, you were referring people with families to support groups or psychologists back then, but it was too much. So, I said ‘I needed to look for a professional’, and I was in therapy to take away the emotional pressure of having to provide solutions all the time, all the time, 24 h, because people were left alone, women without families, cases of children where all the older relatives died. So, there were extreme cases. And yes, I did raise my hand and looked for a therapist because I said, ‘it’s too much, I cannot’. And yes, it works! It really is something good and recommendable” (promotora, focus group).

### Specific characteristics of the training sessions that could help support *promotores*

Key actors and *promotores* provided suggestions and requests to improve the ongoing mental health training sessions. Topics suggested by key actors included communication skills, active listening, interviewing techniques, developing new partnerships, and identifying mental health risks of users, as well as training sessions that included clinical practice opportunities.

“I was just telling you about these skills that are priorities that have to do with awareness, and later essential skills, communication and listening, establishing this environment of trust, so that the person feels heard, and shares with us what is happening” (key actor, semi-structured interview).

*Promotores* suggested crisis response, suicide prevention, schizophrenia, child rearing styles, and gender violence.

“Maybe schizophrenia or suicide. These two, because we almost always focus on what is very common: anxiety, depression, bipolar disorder. I have been at the Ventanilla de Salud for 8 years, and I have taken all the courses that UNAM has offered. So it’s been excellent. I mean, I congratulate them; they have been terrific, but it seems to me that suicide and schizophrenia have been left a little to the side. Personally, I have not had many of these cases, but yes, it’s not like none have ever appeared, and I would like to be a little more prepared to deal with them in case I need to help someone” (health promoter, group focal).

They requested additional training in self-care and additional support tools for themselves as providers. They mentioned that activities such as group and individual self-care guided sessions provided by psychologists, counselors, or other mental health professionals for *promotores* would be very beneficial for their mental health. The most frequently suggested training session frequency for these activities was every 15 days, although others mentioned that, because their time is limited, once a month or every 2 months would be better. Also, they highlighted that sessions should be preferably online during VDS working hours (because many *promotores* have other jobs) or as lunch and learns. However, some *promotores* expressed concerns that the high flow of people in their VDS would not allow them to attend the sessions.

## Discussion

In this study, we explored capability, opportunity, and motivation for *promotores* to provide mental health services during the ongoing health promotion and prevention programming that they routinely conduct through the network of Mexican Consulates in the US. We found that the leadership of the VDS had already implemented some mental health programming, had established partnerships in Mexico with the psychology school of UNAM, and provided training and support for *promotores*. Through the interviews with key actors and the focus groups with *promotores*, we identified strengths of the ongoing program and opportunities to improve it and to fully integrate mental health promotion and prevention into the current services provided through the VDS.

The main strengths found in terms of capability were that *promotores* have at least basic knowledge of the importance of mental health as part of overall health and well-being, and that there are systems in place to monitor the implementation of the mental health strategy and provide feedback. Conversely, there is high staff turnover, which has resulted in many *promotores* without all the skills that have been taught through the training sessions. Also, some *promotores* feel overwhelmed with all the tasks required of them and are not able to pay attention to detail when providing mental health services. Interventions that can help improve capability in this context include additional training, role modeling from more experienced *promotores*, and adding prompts or cues to remind the *promotores* to conduct the mental health questionnaires (19).

Social opportunity was identified as an important area to strengthen the implementation of the intervention. *Promotores* identified different sources of social support including other *promotores*, the VDS leadership, and psychologist form UNAM; however, they also expressed the importance of channeling that support through official and structured channels. They requested periodic support groups where they can talk to each other, express their feelings, and exchange ideas and approaches. This is in line with recommendations from a recent review of health provider mental health during the COVID-19 pandemic that found that healthcare workers relied on social support and contact to address mental health problems (20).

Another important aspect of social opportunity was the big sense of commitment among *promotores* to provide mental health services. They have witnessed firsthand the difficulties faced by VDS users and have a service mindset that compels them to provide these mental health services. However, some participants questioned if the proposed activities go beyond the professional role and training of the *promotores* who are not licensed mental health professionals. Recommendations to address this issue include increasing the partnerships with trained mental health professionals who can provide treatment, and delineating very clearly what is expected from the *promotores* during the VDS work, which is primarily health promotion, screenings, and referrals.

Under certain circumstances, being able to provide support has been deemed positive for providers’ mental health and well-being (21, 22). Inagaki and Orehek identified two conditions that need to be met for providing support to be beneficial for the provider: that support is given by choice and that the individual giving support believes the support is effective (23). In the case of VDS *promotores*, the first condition is met. However, a challenge identified for reflective motivation was that, even though providers feel that their work can have a big impact, they often do not know what happens to the VDS users they refer: whether they are ever contacted by the psychologists and receive the attention they need. This not only could decrease the benefits that they get as providers of support, but also dampens their motivation to conduct screenings and referrals.

Perhaps the most important challenge identified for automatic motivation was the emotional burden that *promotores* experienced while providing mental health services, especially during the COVID-19 pandemic when the mental health vulnerable Latino groups was particularly impacted (24). *Promotores* described experiencing strong emotions while providing mental health services to VDS users, including anxiety, sadness, and frustration, and that sometimes, these emotions and concerns persisted at home and affected their personal lives. Self-care training was implemented by the VDS leadership and the UNAM psychology team as a response to this situation. Self-care was described as extremely helpful by *promotores*, which is consistent with other studies that have improved emotional burnout of community health workers with self-care interventions (25, 26).

High turnover was identified as an important challenge for capability, opportunity, and motivation to implement the mental health services in the VDS. This challenge is potentially related to the emotional burnout associated with proving services to a highly minoritized population, (27, 28) as well as to the limited economic compensation and high demands the VDS *promotores* face. Recommendations are to continue supporting and addressing the mental health needs of the *promotores*, to find additional economic resources and improve economic compensation as much as possible, and to innovate to try to reduce competing tasks and demands. In this sense, most recommendations identified through this study relate to the importance of investing in the well-being of VDS *promotores*. The VDS is a unique program reaching and addressing the preventive needs of thousands of Mexican immigrants in the US and, within this program, *promotores* are a unique workforce that is essential to achieving the goals of the program.

Limitations of this study include that some key actors and *promotores* were not able to participate in the study due to the demanding nature of their jobs, and that all data collection was conducted online which can affect the way people interact, especially in focus groups. However, we were able to collect qualitative data from a diverse group of key actors and *promotores* describing different aspects of the VDS mental health strategy. The use of the COM-B model to explore barriers and opportunities is also a strength of this study, which will allow us to translate the results into actionable solutions that improve the implementation of the VDS mental health strategy and improve mental health prevention for Mexican immigrants.

## Data availability statement

The original contributions presented in the study are included in the article/supplementary material, further inquiries can be directed to the corresponding author.

## Ethics statement

The studies involving humans were approved by Indiana University Bloomington Institutional Review Board (2009002576). The studies were conducted in accordance with the local legislation and institutional requirements. The ethics committee/institutional review board waived the requirement of written informed consent for participation from the participants or the participants’ legal guardians/next of kin because Research was considered exempt as part of a program evaluation. Verbal assent was provided by participants.

## Author contributions

IG: Conceptualization, Data curation, Formal analysis, Funding acquisition, Investigation, Project administration, Supervision, Writing – original draft. DM: Data curation, Formal analysis, Investigation, Writing – original draft. JO: Data curation, Formal analysis, Investigation, Writing – original draft. MR: Conceptualization, Resources, Supervision, Writing – review & editing. MG: Investigation, Supervision, Writing – review & editing. AF: Conceptualization, Funding acquisition, Supervision, Writing – review & editing.
